# Supplementary material for: Comparative multiomics analysis of cell physiological state after culture in a basket bioreactor
Source: Sci Rep. 2022 Nov 23;12:20161. doi: 10.1038/s41598-022-24687-4 (PMC9686226; doi:10.1038/s41598-022-24687-4)
Supplement: Supplementary file 1 — Supplementary Information 1. [file 41598_2022_24687_MOESM1_ESM.zip › raw data/Proteomic raw data/6.KEGGenrichment/EG.vs.CG/UP/pathway/src/00270.html]

map00270


- K00558, K17398, K17399, 2.1.1.37, R04858"

- Up regulated proteins

A0A0D9R4V8(Description:DNA (cytosine-5)-methyltransferase;Gene:DNMT1;log2FC:1.2372)

Close
